# Factors associated with maternal mortality in Kazakhstan: a pre- and during-pandemic comparison

**DOI:** 10.3389/fpubh.2024.1337564

**Published:** 2024-06-03

**Authors:** Karina Nukeshtayeva, Gaukhar Kayupova, Nurbek Yerdessov, Zhanerke Bolatova, Olzhas Zhamantayev, Anar Turmukhambetova

**Affiliations:** School of Public Health, Karaganda Medical University, Karaganda, Kazakhstan

**Keywords:** maternal mortality, global health, health determinants, Central Asia, Kazakhstan, COVID-19

## Abstract

**Introduction:**

The maternal mortality indicator serves as a crucial reflection of a nation’s overall healthcare, economic, and social standing. It is necessary to identify the variations in its impacts across diverse populations, especially those at higher risk, to effectively reduce maternal mortality and enhance maternal health. The global healthcare landscape has been significantly reshaped by the COVID-19 pandemic, pressing disparities and stalling progress toward achieving Sustainable Development Goals, particularly in maternal mortality reduction.

**Methods:**

This study investigates the determinants of maternal mortality in Kazakhstan from 2019 to 2020 and maternal mortality trends in 17 regions from 2000 to 2020, employing data extracted from national statistical reports. Stepwise linear regression analysis is utilized to explore trends in maternal mortality ratios in relation to socioeconomic factors and healthcare service indicators.

**Results:**

The national maternal mortality ratio in Kazakhstan nearly tripled from 13.7 in 2019 to 36.5 per 100,000 live births in 2020. A remarkable decrease was observed from 2000 until around 2015 with rates spiked by 2020. Significant factors associated with maternal mortality include antenatal care coverage and the number of primary healthcare units. Additionally, socioeconomic factors such as secondary education enrollment and cases of domestic violence against women emerged as predictors of MMR. Moreover, the impact of the pandemic was evident in the shift of coefficients for certain predictors, such as antenatal care coverage in our case. In 2020, predictors of MMR continued to include secondary education enrollment and reported cases of domestic violence.

**Conclusion:**

Despite Kazakhstan’s efforts and commitment toward achieving Sustainable Development Goals, particularly in maternal mortality reduction, the impact of the COVID-19 pandemic poses alarming challenges. Addressing these challenges and strengthening efforts to mitigate maternal mortality remains imperative for advancing maternal health outcomes in Kazakhstan.

## 1 Introduction

Mothers’ and children’s health are important measures of a nation’s well-being. The maternal mortality ratio (MMR) reveals performance of a nation’s healthcare system and its economic and social levels (1–3). The MMR is a valuable measure of the quality and organization of modern obstetrical care. The World Health Organization defines maternal mortality as “the number of maternal deaths during a given time period per 100,000 live births during the same time period.” Whereas maternal death is defined as “the death of a woman while pregnant or within 42 days of termination of pregnancy, irrespective of the duration and the site of the pregnancy, from any cause related to or aggravated by the pregnancy or its management, but not from accidental or incidental causes” (4).

Goal 3 (SDG 3) of the UN’s Sustainable Development Agenda calls for a reduction in the worldwide MMR to fewer than 70 per 100,000 live births by 2030. The pandemic of COVID-19 has had a substantial adverse influence on maternal health and the progress toward reducing MMR. It has exacerbated existing disparities in healthcare access, particularly in developing countries (5, 6). Studies have revealed a substantial rise of maternal mortality at the time of the pandemic compared to pre-COVID period, even though measuring maternal mortality during pandemics can be challenging. Some results demonstrate a significant difference between high- and low-resource environments (7, 8). The impact of COVID-19 is far-reaching and influenced by various factors such as climate, societal behavior, human activities, governance, economy, and technology. To meet the goal of lowering maternal mortality and enhancing maternal health, it is critical to understand the distribution of these effects across different populations, particularly those who are more vulnerable, and address the various factors that contribute to the problem (9).

Nearly 95% of all maternal deaths in 2020, according to the WHO, took place in countries with low and lower middle incomes (10). In 2017, the MMR were 462 and 11 per 100,000 live births in low-income and high-income countries, respectively (11). Maternal mortality rates remain high in the least developed nations of the world, approximated at 415 maternal deaths per 100,000 live deliveries, more than 40 times greater than in Europe (12). According to UN inter-agency estimates the MMR decreased by 34% between 2000 and 2020, from 342 deaths to 223 deaths per 100,000 live births, indicating considerable success in lowering maternal mortality on a global scale (13). However, 92% of the 129 countries surveyed at the end of 2021 had their health services disrupted by COVID-19, resulting in a halt in progress toward universal health coverage (14).

Maternal mortality is influenced by several contextual factors including age, marital status, the number of antenatal visits, education level, divorce rates, lack of health workforce and others (15, 16). Despite significant socioeconomic development over the last two decades and the introduction of consecutive health programs aimed at improving primary care, particularly maternal health, Kazakhstan still trails behind Organisation for Economic Co-operation and Development (OECD) countries in leading health indicators (17). Healthcare expenditure in Kazakhstan saw a yearly increase from 2011 to 2020, the nation’s healthcare system still suffers from substantial underfunding. Consequently, household expenditures significantly contribute to overall health spending in Kazakhstan (18).

Over the past couple of years, there’s been a surge in domestic violence incidents in Kazakhstan. The majority of victims are women, making up over 77.9% of cases. The most vulnerable groups include women aged between 30–39 (37.80%) and 40–49 (41.46%) (19). In terms of education, according to a 2014 OECD study, 28% of women pursued higher education compared to 23% of men (20). Moreover, both women and men in Kazakhstan have extremely high literacy rates (99.9%). The labor force participation rate stands at 63.2% for women and 74.5% for men (21). In Kazakhstan, the percentage of births attended by skilled health personnel has been around 99.9% for the last decade, significantly higher than the global average of 86.2% in 2022 (22). All of these, which could be considered as contextual and structural factors, may contribute to maternal mortality and health. Therefore, they should be examined in this context.

In this research, we aimed to analyze the determinants which contributed to the maternal mortality in Kazakhstan between the period of 2019 to 2020.

## 2 Materials and methods

### 2.1 General information about the Republic of Kazakhstan

The study was conducted in Kazakhstan. It is a landlocked middle-income country in the center of Eurasia, with a small amount of its area belonging to Europe and the majority of it being Asia. The population of Kazakhstan is estimated at 19,644,067 by the State Statistics Committee (August 1, 2022). The area of the territory is 2,724,902 km^2^. By territory, it holds the ninth-place position in the globe. Kazakhstan borders China, Russia, Kyrgyzstan, Uzbekistan, and Turkmenistan (23). Administratively, the Republic of Kazakhstan is split into 14 regions and three significant republican cities (Astana, Almaty, and Shymkent).

### 2.2 Data sources

The work of healthcare institutions and the entire population of Kazakhstan in the period from 2000 to 2020 was studied using data from the annual statistical report (24). These reports include information on population health and health care services at the national and regional levels. To ensure comparability of statistics, health data collection practices in Kazakhstan comply with international standards. Socioeconomic data of the population was obtained from the official statistical portals of the Republic of Kazakhstan.

In this study, we analyzed several key factors sourced from the annual report. These factors included healthcare service and socioeconomic predictors.

#### 2.2.1 Healthcare service factors

Antenatal care coverage serves as a measure of the accessibility and utilization of healthcare services throughout pregnancy (25).

The volume of healthcare services provided refers to the total cost of healthcare services provided by organizations, which is derived from funds received from enterprises, organizations, and individuals (households) in exchange for those services (26).

Abortions per 100 live births, including stillbirths, is the total number of induced abortions, regardless of the method employed (27).

Number of primary health care units encompasses all healthcare facilities offering outpatient services, such as hospital outpatient departments, polyclinics, ambulatory centers, medical clinics, and medical aid posts (28).

Nursing and midwifery personnel (per 10,000 population) refers to the number of nursing and midwifery professionals per 10,000 people in the population (29).

Obstetricians and gynaecologists, per 10,000 population is a measure the density of obstetricians and gynecologists within a given population (30).

#### 2.2.2 Socioeconomic factors

Gross Domestic Product (GDP) *per capita* is an economic indicator that measures the average wealth generated per person in a country (31).

The poverty rate is a measure that represents the proportion of individuals (typically within a specific age group) whose income levels are below the designated poverty line (32).

Average age of the population is a statistical measure that represents the central tendency of ages within a population (33).

Marriage rate is a demographic indicator that measures the number of marriages occurring within a specific population during a given year (34).

Female labor force is a demographic indicator that measures the proportion of the female population within this age group who are economically active (35).

Total divorce ratio is a metric derived from the number of divorces finalized within a single year.^1^

Secondary education enrollment (11–17 years), % represents the proportion of total enrollment, irrespective of age, relative to the population within the specified age group corresponding to the indicated educational level (36).

Number of reported cases of domestic violence against women is the number of women who believe that a husband or partner is justified in physically assaulting his wife or partner under specific circumstances (37).

### 2.3 Statistical analysis

We conducted a comprehensive examination of the maternal mortality ratio in Kazakhstan across all 14 regions and 3 cities of republican significance spanning a 20-year timeframe. Our study investigated the relationships between MMR and various socioeconomic and healthcare service variables both before and during the COVID-19 pandemic, employing a both-direction Stepwise Linear Regression analysis. Stepwise Linear Regression was utilized to identify the optimal subset of explanatory variables for multiple regression models for the years 2019 and 2020. This method involves comparing improvements in the Akaike Information Criterion by systematically removing or adding candidate variables within the specified bounds of regressors. We identified a total of 78 variables based on a thorough review of literature and data availability pertaining to the determinants of MMR in Kazakhstan during the aforementioned years. These variables were categorized into two distinct groups: Socioeconomic and Healthcare Service factors, leading to the derivation of four multiple regression models:

MMR for the 2019-year (Group of Healthcare service indicators) = α + β1* obstetricians and gynaecologists, per 1,000 born + β2* Antenatal care coverage, including early register of pregnancy up to 12 weeks (%) + β3* Medical examination of the population per 100,000 population + β4* Abortions per 100 live births, including stillbirths + β5* Number of primary health care units + β6* Nursing and midwifery personnel per 10,000 population.
MMR for the 2019-year (Group of Socioeconomic predictors) = α + β1* GDP *per capita*, in dollars + β2* poverty rate + β3* Average age of the population + β4* Total divorce ratio + β5* Gross enrollment in secondary education (11–17 years) % + β6* Gross coverage of higher education (18–22 years) % + β7* Number of reported cases of domestic violence against women.
MMR for the 2020-year (Group of Healthcare indicators) = α + β1* Number of Obstetricians per 1,000 born + β2* Antenatal care coverage, including early register of pregnancy up to 12 weeks(%) + β3* medical examination of the population per 100,000 population + β4* Abortions per 100 live births, including stillbirths + β5* Number of primary health care units + β6* Nursing and midwifery personnel per 10,000 population + β7* obstetricians and gynaecologists, per 1,000 born.
MMR for the 2020-year (Group of Socioeconomic predictors) = α + β1* poverty rate + β2* femalealbor force (aged 15 and over) + β3 * marriage rate + β4* Total divorce ratio + β5* Gross enrollment in secondary education (11–17 years) % + β6* Gross coverage of higher education (18–22 years) % + β7* Number of reported cases of domestic violence against women.

The coefficients of statistically significant variables derived from these models are outlined in Tables 1, 2.

**Table 1:** Factors associated with MMR before pandemic (2019).

Predictor variable	Coefficient	Standard error	*t*-value	*p*-value
Healthcare service indicators				
Intercept	245.324	49.925	4.914	0.045
Antenatal care coverage, including early register of pregnancy up to 12 weeks, %	−2.513	0.588	−4.273	0.003
The volume of healthcare services provided	0.129	0.033	3.912	0.005
Abortions per 100 live births, including stillbirths	1.000	0.176	5.670	0.001
Number of primary health care units	0.043	0.015	2.833	0.02
Nursing and midwifery personnel (per 10,000 population)	−0.891	0.165	−5.403	0.001
Model summary: R2 0.9256/ Adjusted R2 0.851/ MSE 7.54				
Socioeconomic indicators				
Intercept	178.407	64.805	2.753	0.028
Total divorce ratio	14.964	5.932	2.523	0.03
Enrollment in secondary education (11–17 years), gross %	−1.461	0.436	−3.354	0.01
Number of reported cases of domestic violence against women, 1,000	1.528	0.459	3.323	0.01
Model summary: R2 0.86/ Adjusted R2 0.7201/ MSE 14.18				

**Table 2:** Factors associated with MMR during pandemic (2020).

Predictor variable	Coefficient	Standard error	*t*-value	*p*-value
Healthcare indicators				
Intercept	690.649	171.124	4.036	0.00496
Antenatal care coverage, including early register of pregnancy up to 12 weeks	−9.464	2.533	−3.736	0.00730
Number of primary health care units	−0.215	0.084	−2.563	0.03738
Model summary: R2 0.7959/ Adjusted R2 0.5335/ MSE 10.43				
Socioeconomic indicators				
Intercept	414.879	91.353	4.542	0.00107
Gross enrollment in secondary education (11–17 years) %	−2.399	0.861	−2.786	0.01924
Number of reported cases of domestic violence against women, 1,000	6.038	3.173	1.903	0.04623
Model summary: R2 0.7006/ Adjusted R2 0.5209/ MSE 15.13				

We also described the MMR trends in Kazakhstan regions from 2000 to 2020. For statistical analysis, we applied IBM SPSS Statistics version 26.

## 3 Results

### 3.1 MMR and its trends

**Figure 1:**
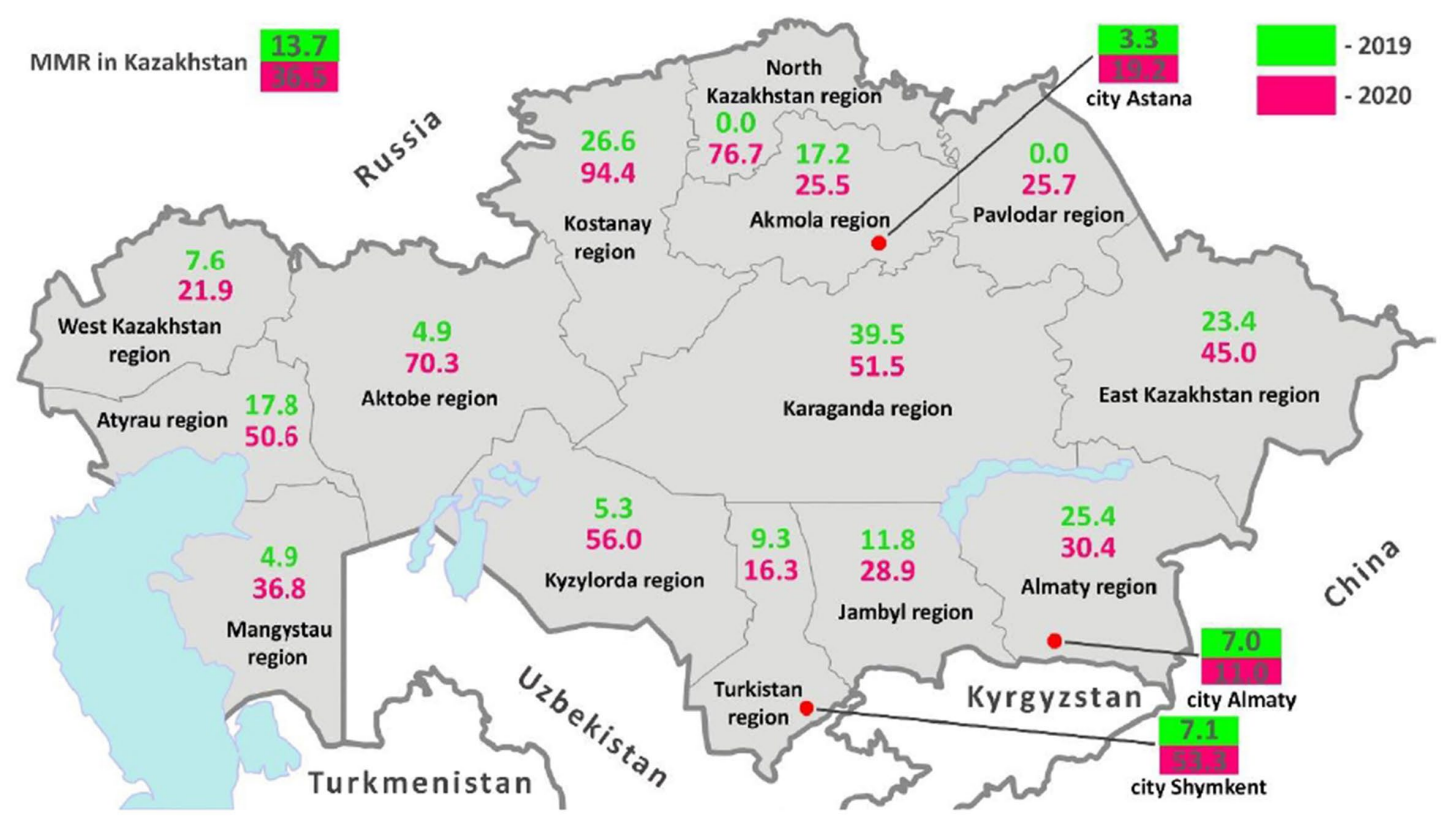
MMR before and during COVID-19 in Kazakhstan.

In 2020, the national MMR almost tripled compared to 2019 (13.7 in 2019 and 36.5 per 100,000 live births in 2020). Also, there was a dramatic increase of MMR in 2020 in all regions of Kazakhstan. Thus, in the North Kazakhstan and Pavlodar regions, where no cases of maternal mortality were registered in 2019, during the pandemic there was a sharp rise in this indicator to 76.7 and 25.7 per 100,000 live births, respectively. In terms of MMR, the Karaganda region led in 2019 with an indicator of 39.5 per 100,000 live births, while the Kostanay region led in 2020 with an indicator of 94.4 per 100,000 live births (Figure 1).

**Figure 2:**
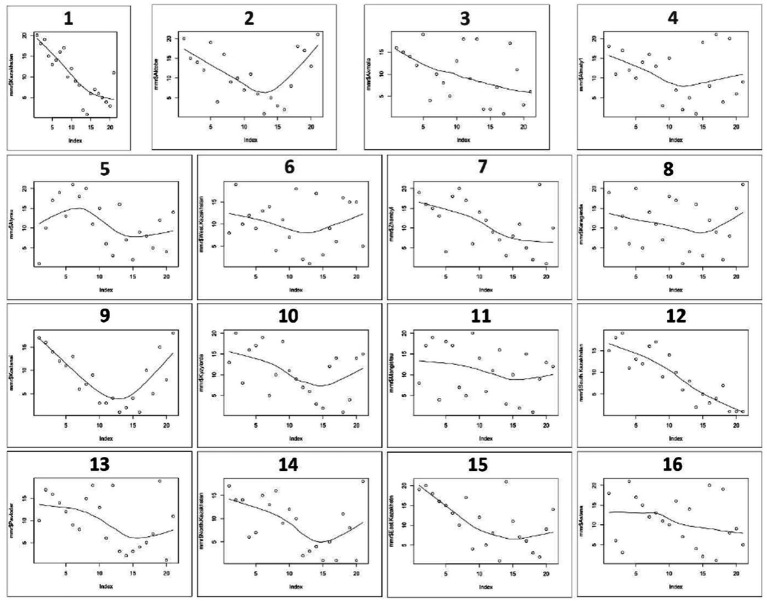
MMR trends in Kazakhstan Regions from 2000 to 2020. 1 – Kazakhstan, 2 – Aktobe region, 3 – Akmola region, 4 – Almaty region, 5 – Atyrau region, 6 – West Kazakhstan Region, 7 – Zhambyl region, 8 – Karaganda region, 9 – Kostanay region, 10 – Kyzylorda region, 11 – Mangistau region, 12 – South Kazakhstan region, 13 – Pavlodar region, 14 – North Kazakhstan region, 15 – East Kazakhstan region, 16 – Astana city.

MMR trends in various parts of Kazakhstan indicate a consistent fall beginning in 2000 and continuing until 2010–2015, then increasing by 2020. Maternal mortality per 100,000 live births has dramatically increased after 2010–2015 in the Aktobe region (from 10 in 2015 to 70.3 in 2020), in the Almaty region (from 5.1 in 2013 to 30 0.4 in 2020), West Kazakhstan region (from 7.9 per in 2013 to 21.9 per in 2020), Karaganda region (from 4.1 in 2013 to 39.5 in 2020), Kostanay region (from 15.3 in 2013 to 94.4 in 2020), Kyzylorda region (from 10 in 2014 to 56 in 2020), North Kazakhstan region (from 12.1 in 2011 to 76.7 in 2020) (Figure 2).

### 3.2 Factors associated with the MMR before and during pandemic

The dependent variable of the linear regression was MMR, with a mean of 12.91 and a standard deviation of 10.88, the MMR values ranged from 0 to 39.50 for 2019 (Table 3).

**Table 3:** Descriptive statistics for indicators before pandemic (2019).

Variable	Mean	Standard deviation	Minimum	Maximum
Healthcare service indicators				
Antenatal care coverage, including early register of pregnancy up to 12 weeks, %	88.20	2.47	84.30	92.30
The volume of healthcare services provided, billion teenage	83.23	57.19	40.21	235.69
Abortions per 100 live births, including stillbirths	22.64	11.21	6.70	50.10
Number of primary health care units	115.00	76.57	28.00	302.00
Nursing and midwifery personnel (per 10,000 population)	53.15	10.92	37.60	75.90
Socioeconomic indicators				
Total divorce ratio	3.26	0.71	1.76	4.24
Secondary education enrollment, gross %	99.9	0	99.9	99.9
Number of reported cases of domestic violence against women, 1,000	5.66	5.68	1.52	25.88
Outcome				
MMR per 100,000	12.91	10.88	0.00	39.50

The descriptive statistics for variables of linear regression showed, that 88.20% of pregnant women received antenatal care, with a standard deviation of 2.47%. The volume of healthcare services provided ranged from 40.21 to 235.69 billion tenge and showed a mean of 83.23 with a standard deviation of 57.19 billion tenge. Abortions per 100 live births, including stillbirths, ranged from 6.70 to 50.10, with a mean rate of 22.64. The average availability of primary healthcare units indicated 115 units with a standard deviation of 76.57, suggesting differing levels of healthcare accessibility across regions. The density of nursing and midwifery personnel per 10,000 population averaged 53.15.

According to socioeconomic indicators, the mean divorce ratio was 3.26 thousand with a deviation of 0.71. The average gross enrollment rate in secondary education was 99.9%. The indicator “violence against women and girls” averaged 5.66, with a standard deviation of 5.68.

Table 1 illustrates the factors exerting a statistically significant influence on the MMR in Kazakhstan during the pre-COVID period. Specifically, healthcare service indicators such as the antenatal care coverage (*β* = −2.51, *p* < 0.01) and the availability of nursing and midwifery personnel per 10,000 population (*β* = −0.89, *p* = 0.001) demonstrate associations with a reduction in the MMR.

An analysis of socioeconomic factors as predictors of MMR reveals notable associations in Kazakhstan for the year 2019. Specifically, divorce ratio (*β* = 14.96, *p* < 0.05) is linked with a significant rise in MMR, while a higher percentage of gross enrollment in secondary education correlates with a reduction in MMR. Additionally, it is noteworthy that instances of domestic violence against women (*β* = 1.53, *p* = 0.01) contribute to an elevated MMR.

The output of linear regression indicated as MMR during pandemic time, and the average MMR was approximately 41.97 deaths per 100,000 live births, with a standard deviation of 23.27 (Table 4). On average, about 85.1% of pregnant women receive antenatal care, including early registration, up to 12 weeks, with a standard deviation of 4.11%. Moreover, this indicator ranged from 76.40 to 91.80%. The average number of primary health care units available in the population was approximately 186.41 units, with a wide standard deviation of 90.41. Almost all population within the age group of 11–17 years are enrolled in secondary education. The number of reported cases of domestic violence against women varied from 1.14 to 13.02, also the mean was approximately 3.72, with standard deviation of 2.92.

**Table 4:** Descriptive statistics for indicators during the pandemic (2020).

Variable	Mean	Standard deviation	Minimum	Maximum
Healthcare indicators				
Antenatal care coverage, including early register of pregnancy up to 12 weeks, %	85,1765	4,11,180	76,40	91,80
Number of primary health care units	186,4,118	90,41,782	48,00	370,00
Socioeconomic indicators				
Enrollment in secondary education (11–17 years), gross %	99.9	0	99.9	99.9
Number of reported cases of domestic violence against women, 1,000	3,7,187	2,92,542	1,14	13,02
Outcome				
MMR per 100,000	41,9,706	23,27,031	11,00	94,40

A comprehensive regression analysis focusing on healthcare indicators pertaining to MMR unveils two statistically significant factors linked with a reduction in MMR: the number of primary health care units (*β* = −0.21, *p* < 0.01) and the antenatal care coverage, including early register of pregnancy up to 12 weeks (*β* = −9.46, *p* < 0.01). Furthermore, it is noteworthy that the coefficient for the predictor “Antenatal care coverage, including early register of pregnancy up to 12 weeks, %” experienced a notable shift during the pandemic period, dropping to −9.46 from −2.51 observed in the pre-pandemic period.

Similar to the findings in 2019, predictors of MMR in 2020 include the percentage of gross enrollment in secondary education (11–17 years) (*β* = −2.39, *p* < 0.05) and number of reported cases of domestic violence against women. Also, in 2020, the surge in domestic violence cases is significantly correlated with a notable rise in the average MMR in Kazakhstan (*β* = 6.04, *p* < 0.05).

## 4 Discussion

Our research presents the first attempt to identify and compare the healthcare and socioeconomic factors influencing MMR across different regions of Kazakhstan before and during the COVID-19 pandemic. Furthermore, our comparison reveals how the pandemic affected maternal health differently, giving important insights for targeted actions and policy adjustments during global health crises.

According to the results of this study, MMR patterns in many Kazakhstani regions showed a consistent reduction from 2000 to 2010–2015, followed by an increase by 2020, which is partially in line with the global trend of the decrease in MM during the same time period. It can be noted that in 2020 the MMR in Kazakhstan increased almost threefold compared to the previous year. Additionally, there was a sharp jump in some regions from 0 to 25.7 and even up to 76.7 per 100,000 live births in 2020. During the last 20 years, all Kazakhstani regions had MMR fluctuations except Akmola region, South Kazakhstan, and Astana city.

Furthermore, MMR was linked to measures of population health, socioeconomic status, and health services. Most of these indicators were negatively associated with MMR, while number of reported cases of domestic violence against women, total divorce ratio, the volume of healthcare services provided, abortions were positively associated with MMR. Number of primary HC units was positively associated with MMR in 2019 and negatively associated with MMR in 2020 (Tables 1, 2).

### 4.1 Interpretation (in light of other evidence)

The MMR and socioeconomic, medical, and morbidity indicator factors were shown to be significantly correlated in our study. Our analysis revealed several key determinants influencing MMR: socioeconomic factors (total divorce ratio, enrollment in secondary education, number of reported cases of domestic violence against women), healthcare related factors (volume of healthcare services provided, abortion rates, amount of nursing and midwifery personnel, number of primary health care units, antenatal care coverage). These findings align with the literature describing the individual-level factors (age, parity), household characteristics (location, access to clean water), community elements (socioeconomic status), and systemic aspects (healthcare facilities) as prominent influences on maternal mortality (2, 3, 38).

Since 2000, Kazakhstan’s MMR was the lowest among Central Asian nations, and it had been declining for the previous 10 years. This achievement can be attributed to the realization of the Government policy of the Republic of Kazakhstan, formulated through the consistent government programs: “Salamatty Kazakhstan” (2011–2015), “Densaulyk” (2016–2019), The Concept of healthcare development of the Republic of Kazakhstan until 2026 (39). Considering the challenges the nation’s healthcare system faces, the primary strategies have been established in a number of areas, with one of the top goals being the improvement of women’s and children’s health.

According to the Multiple Indicator Cluster Survey in the Republic of Kazakhstan, 2010–2011 99.2% of pregnant women received antenatal care at least once throughout their pregnancy, indicating a very high coverage rate for this type of care (40). In the research conducted by Dauletyarova et al. (41) 90.0% of the women expressed satisfaction with the antenatal care they received. Our research findings identified that 88.2% of pregnant women were covered with antenatal care services in 2019, whereas in 2020 this indicator dropped to 85.17%. Regression analysis revealed that the coverage of antenatal care had a higher impact on the decline in MMR in 2020 compared to 2019 (Tables 1,2).

Due to difficulties in obtaining healthcare, variations in pandemic containment strategies, and a high frequency of COVID-19 risk factors there may be an increased risk of maternal deaths as a result of COVID-19 in low- and middle-income countries affected by the pandemic (7, 42). El-Shal et al. (43) suggests that health emergencies, such as the COVID-19 pandemic, can instantly increase maternal, under-five, and neonatal mortality by 0.3, 0.3, and 0.2% and, after 1 year by 35, 80, and 26%, respectively (44). Our findings show that while Kazakhstan experienced a general decline in MMR from 2000 through around 2015, there was an alarming increase by 2020. In contrast to 2019, the MMR rapidly climbed in 2020, rising by 2.6 times to 36.5 per 100,000 live births (45). A nearly threefold rise in MMR nationally from 2019 to 2020 suggests significant systemic stressors at play. The growth of excess mortality is being observed around the world, and the pandemic contributed to it. The study from Brazil reported that, even after accounting for the anticipated excess mortality from COVID-19, the excess maternal mortality in Brazil in 2020 was 1.40 (95% CI 1.35–1.46) (46). The pandemic also resulted in a 33.3% relative rise in maternal fatalities in the US (47). Furthermore, in the USA the number of maternal mortality cases grew with maternal age (43). A study from Kazakhstan reported that higher age associated with greater mortality risk in this period, and from 2019 to 2021 in Kazakhstan, there was a rise in mortality rates among women aged 35–39 from 0.48 to 4.37 per 100,000 individuals (48, 49). Overall, even before the pandemic, obesity and high school non-completion were identified as important contributors to maternal mortality, while access to healthcare services and resources, family factors, such as revenue and education, and ecological determinants as poverty, gender inequality, human development also played a role (3, 6, 50).

Our linear regression analysis showed that factors such as antenatal care coverage, percentage of population enrolled in secondary education, number of nursing and midwifery personnel, were statistically significantly associated with a decreased level of MMR. These findings contribute to the existing body of literature by providing specific insights into the Kazakhstani context. Thus, increasing coverage of midwife-delivered interventions potentially significantly influence the reduction of stillbirths, neonatal fatalities, and maternal mortality. 41% of maternal deaths, 39% of neonatal deaths, and 26% of stillbirths might be avoided with a significant increase in coverage. Universal coverage has the potential to make the largest impact in low- to medium-HDI countries, potentially saving up to 4.3 million lives yearly by 2035 (51). The decrease in the availability of competent healthcare professionals during childbirth due to reductions in government health spending has been found to significantly contribute to an increase in maternal mortality rates (52). A well-equipped, empowered, and effectively deployed healthcare workforce is critical for the attainment of health-related SDGs. Most high-income countries have an adequate number of health professionals to provide necessary treatment, and they generally have strong educational and regulatory systems in place. Similar trends can also be observed in upper-middle-income countries, with their policies being supportive of the healthcare sector (53–55). The Universal Health Coverage (UHC) Index for Kazakhstan has seen a steady increase from 2000 to 2021, which can be considered as a positive indicator in the context of maternal mortality. Starting at a score of 56 in 2000, the index rose gradually over two decades to reach and maintain a score of 80 by 2019 through 2021. Improvements in the UHC suggest that more people in Kazakhstan are accessing essential health services, which includes maternal and child healthcare (56). Moreover, since universal health coverage is aligned with SDG 3.8.1, which specifically aims to achieve equity in healthcare services including reproductive, maternal, newborn, and child health, this upward trajectory correlates with efforts directed toward lowering the MMR (57). Since the mid-2000s, Kazakhstan has established youth health centers that play a significant role in enhancing maternal health among adolescents. These centers provide young women with education on prenatal care, which is essential for reducing pregnancy-related complications and maternal mortality. These centers strive to prevent unsafe abortions by guiding adolescents toward effective family planning methods and contraceptive use, thereby reducing unwanted pregnancies and associated health risks (58). In the context of maternal health and mortality, Kazakhstan’s fall to 72nd place in gender equality list in 2019 highlights significant disparities affecting women’s health and survival. The country’s decline from 32nd since 2006 signals pressing issues like wage gaps, few women leaders, and unequal domestic workloads. Pregnant women and mothers face severe discrimination that can hinder job access and growth, leading to financial issues which negatively impact maternal healthcare availability and increase mortality risks (59). Central Asian Sample Survey on the prevalence of violence against women indicated a significant incidence of domestic abuse in Kazakhstan. Of the ever-partnered women aged 18–75 surveyed, 17% reported encountering physical or sexual violence from a partner, while 21% faced psychological abuse (60). Domestic violence had a larger influence on the rise in MMR in 2020 than it did in 2019, according to our data, even though the number of instances fell from 5.66 thousand in 2019 to 3.718 thousand in 2020. Recently, the Kazakhstan government has enacted a New Law on the Rights of Women and Safety of Children. This law aims to challenge male chauvinism embedded within society and legal frameworks by advocating for legislation that historically favors men’s interests at the expense of recognizing and protecting women’s vulnerabilities (61).

In low and low-middle-income countries, maternal education was revealed to be one of the most important individual factors of maternal mortality, along with the location of delivery, delays in seeking health care, prenatal care, and competent birth attendance (62). According to a 2014 OECD study, 28% of women pursued higher education compared to 23% of men (20). Furthermore, both women and men have very high literacy rates (99.9%), and the labor force participation rate in Kazakhstan stands at 63.2% for women and 74.5% for men (21).

While government revenue is crucial, the quality of governance plays an even more key role in achieving positive results for mothers and children. This impact is more pronounced in low-income countries. As government revenue reaches a level of around 5,000 USD *per capita*, the impact of governance on outcomes becomes less critical. The incidence and maternal mortality rates for COVID-19 were higher in the localities with less access to healthcare and greater socioeconomic disparities (63). In Kazakhstan, the correlation between high out-of-pocket healthcare spending and low government health investment is evident. With public healthcare expenditure at merely 2% of the GDP, it falls significantly short of the average 6.5% reported by countries in the OECD (64). Kazakhstan’s recent implementation of a mandatory health insurance system, where the Social Health Insurance Fund became the purchaser of publicly funded health services in 2020, represents a significant shift in healthcare financing. These reforms aim to enhance the accessibility, equity, and efficiency of health services. The introduction of mandatory health insurance complements the state-guaranteed basic package, diversifying funding sources for healthcare. Despite these efforts, public spending on health decreased from approximately 75% in 2009 to around 60% in 2019. Meanwhile, out-of-pocket payments rose to constitute 33.9% of total health expenditure by 2019 (65).

A greater risk of maternal morbidity and mortality exists among pregnant women who have been diagnosed with COVID-19. There are findings showing that COVID-19 during pregnancy leads to a constant and significant increase in severe maternal health issues, mortality, and neonatal complications in comparison to pregnant women who do not have COVID-19. SARS-CoV-2 infection increases the risk of unfavorable pregnancy outcomes, as do pre-existing comorbidities such chronic hypertension, diabetes, advanced maternal age, and high body mass index (66). In Kazakhstan, living conditions and income levels have a significant association with health outcomes (67).

Our study revealed several socioeconomic and healthcare-related determinants associated with the maternal mortality in Kazakhstan in the pre- and during pandemic periods. To enhance the effectiveness of interventions aimed at reducing maternal mortality, it’s essential to systematically address these factors. Given the amount of research showing a correlation between social and economic conditions and maternal deaths, the social and economic components should receive significant attention. Addressing domestic violence prevention and improving access to education and healthcare services are critical steps in this regard. Pregnancy care coverage, the availability of nurseries, and midwifery services are examples of health care determinants that should be guaranteed. Moreover, to inform decision-making and intervention strategies, there’s a pressing need to strengthen scientific research in both public health and clinical medicine. Building a robust evidence base is fundamental for developing effective interventions and improving maternal health outcomes. By systematically addressing socioeconomic and healthcare-related determinants of maternal mortality through targeted interventions and multisectoral collaboration, significant strides towards reducing maternal mortality and improving maternal health outcomes can be done.

### 4.2 Strengths and limitations

Our study presents several strengths, including its comprehensive coverage of regional trends in MMR in Kazakhstan and the use of statistical methods compliant with various studies and publications in the field. We pursued to unravel the role of structural and contextual predictors of MMR. The study also benefits from using officially recognized data sources, which enhances the reliability of our findings.

There are limitations that should be acknowledged. First, as we utilized secondary data obtained from annual statistical reports, there may be inherent biases in the data collection procedure. Given the concerns regarding the potential for biases in data collection and reporting, it is important to highlight that the process of diagnosing, registering, and reporting maternal mortality cases in Kazakhstan is conducted in accordance with rigorous national standards. The Rules for the Provision of Maternal Mortality Information, as outlined by the Ministry of Justice of the Republic of Kazakhstan, ensure that each case of maternal death is thoroughly documented and reported through a structured framework. This legal document is publicly accessible and provide guidelines that dictate how healthcare institutions should report instances of maternal mortality promptly and accurately (68). Additionally, the Standard of Pathological and Anatomical Diagnostics in the Republic of Kazakhstan issued by the Minister of Health further specifies the procedures and protocols for post-mortem examinations. This standard outlines the technical aspects of how maternal deaths are to be diagnosed pathologically (69). It includes criteria for autopsy practices as well as histological examinations when required. These documents collectively establish a comprehensive protocol ensuring that all cases of maternal deaths are not only appropriately diagnosed following medical best practices but also systematically recorded and reported in a manner consistent with legal requirements.

Second, potential confounding variables that might have influenced maternal mortality were not fully explored. Structural and contextual factors such as cultural practices, access to transportation, geographic barriers to care, and personal health behaviors could have significant implications on MMR but were not accounted for in our analysis.

Third, the generalizability of our findings beyond the study sample might be constrained due to unique socioeconomic, cultural, or health system factors present in Kazakhstan that may not be applicable elsewhere.

## 5 Conclusion

Various socioeconomic conditions, aspects of the healthcare system, and the prevalence of diseases significantly impact the complex issue of maternal mortality. Kazakhstan is committed to achieving the Sustainable Development Goals, which include reducing maternal mortality. However, there may have been fluctuations in maternal mortality ratios due to the nation’s middle-income status and the strike of the COVID-19 pandemic. In the broader socio-political context of Kazakhstan, our findings highlight the importance of healthcare infrastructure, socioeconomic factors, and women’s health services in addressing maternal mortality. In light of the significance of socioeconomic and healthcare service-related factors, it is necessary to address population dynamics and health system issues that pose a threat to mothers’ health. The COVID-19 pandemic and global threats such as natural disasters and military conflicts continue to challenge the global healthcare system, further highlighting the need for governments to create a supportive environment that promotes maternal health and reduces mortalities. To effectively tackle maternal mortality, understanding key risk factors as well as determinants that contribute to this issue is important. The reduction of maternal mortality remains a priority for all healthcare systems and public health actors should continuously assess and address these factors to achieve this goal.

## Data availability statement

Publicly available datasets were analyzed in this study. This data can be found at: https://www.gov.kz/memleket/entities/dsm/documents/details/246287?lang=en https://www.gov.kz/memleket/entities/dsm/documents/details/58654?lang=en.

## Author contributions

KN: Writing – original draft, Methodology, Formal analysis. GK: Writing – review & editing, Writing – original draft, Formal analysis, Conceptualization. NY: Writing – original draft, Visualization, Formal analysis. ZB: Writing – original draft, Methodology, Formal analysis. OZ: Writing – review & editing, Writing – original draft, Formal analysis, Conceptualization. AT: Writing – review & editing, Supervision, Project administration.

## Footnotes

^1^https://www.stat.fi/meta/kas/kok_eronneisuus_en.html
